# Hitting the Rewind Button: Imagining Analogue Trauma Memories in Reverse Reduces Distressing Intrusions

**DOI:** 10.1007/s10608-024-10488-8

**Published:** 2024-05-24

**Authors:** Julina A. Rattel, Sarah Danböck, Stephan F. Miedl, Michael Liedlgruber, Frank H. Wilhelm

**Affiliations:** Division of Clinical Psychology and Psychopathology, Department of Psychology, Paris Lodron University Salzburg, Hellbrunner Straße 34, 5020 Salzburg, Austria

**Keywords:** PTSD, Trauma film, Imaginal exposure, Cognitive therapy, Imagery rescripting

## Abstract

**Background:**

Intrusive re-experiencing of trauma is a core symptom of posttraumatic stress disorder. Intrusive re-experiencing could potentially be reduced by ‘rewinding’, a new treatment approach assumed to take advantage of reconsolidation-updating by mentally replaying trauma fast-backward.

**Methods:**

The present analogue study was the first to investigate ‘rewinding’ in a controlled laboratory setting. First, 115 healthy women watched a highly aversive film and were instructed to report film-related intrusions during the following week. Twenty-four hours after film-viewing, participants reporting at least one intrusion (N = 81) were randomly allocated to an intervention (fast-backward, or fast-forward as active control condition) or a passive control condition. Intervention groups reactivated their trauma memory, followed by mentally replaying the aversive film either fast-backward or fast-forward repeatedly.

**Results:**

Results indicate that replaying trauma fast-backward reduced intrusion load (intrusion frequency weighted for intrusion distress) compared to the passive group, whereas replaying fast-forward did not. No above-threshold differences between fast-backward and fast-forward emerged.

**Conclusion:**

Present findings strengthen the view that ‘rewinding’ could be a promising intervention to reduce intrusions.

## Introduction

Intrusions of a traumatic event form a hallmark symptom of posttraumatic stress disorder (PTSD; American Psychiatric Association, 2013). They are easily triggered during daily life by stimuli resembling some aspects of the traumatic event and their intrusiveness makes them highly distressing for patients (Ehlers & Clark, 2000). Therefore, current PTSD treatments aim at reducing these intrusions, though, the often-used lengthy exposure treatment causes high dropout rates, possibly driven by high patient distress (Imel et al., 2013; Lewis et al., 2020). Moreover, exposure-based treatment approaches do not seem to change the existing trauma memory; they merely foster the formation of a competing memory trace, often resulting in treatment benefits declining over time (Bouton, 2014; Vervliet et al., 2013). Therefore, these shortcomings gave rise to other approaches, such as the use of reconsolidation-updating.

Reconsolidation is a process whereby previously consolidated (stable) memories become reactivated by means of a retrieval cue (Merlo et al., 2014), rendering these memories malleable; these memories then require re-stabilization to persist (Nader & Hardt, 2009). It has been proposed that during this time-window, interventions could either enhance or disrupt re-stabilization and thereby update these memories (Lee et al., 2017; Schiller et al., 2010). A promising clinical intervention assumed to work through reconsolidation-updating of emotional memory components is imagery rescripting (Arntz, 2012; see meta-analysis by Morina et al., 2017), used to modify the content of unpleasant memories into more pleasant, less distressing ones (Holmes et al., 2007). For instance, haunting memories of physical assault could be rescripted into memories where the victim successfully disempowers the perpetrator. To gain a better understanding of underlying mechanisms (Morina et al., 2017), researchers frequently use the trauma film paradigm; by showing healthy participants aversive film scenes, researchers can study the development, maintenance, and modification of aversive memories in a controlled environment (James et al., 2016). Using this approach, researchers showed that same-day imagery rescripting resulted in less intrusions during the following days than imagery re-experiencing (c.f. imaginal exposure), positive imagery, or a control condition (Dibbets & Arntz, 2016; Hagenaars & Arntz, 2012). Extending these findings by a 24 h consolidation window following film-viewing, results pointed in the same direction, with imagery rescripting being linked to an accelerated decline in intrusions, though, no group differences in terms of intrusion frequency were found (Siegesleitner et al., 2019). Clearly, more analogue research is needed that takes into account a sufficient consolidation time window to properly study the effectiveness of interventions assumed to work through reconsolidation updating.

‘Rewinding’, a treatment approach suggested long ago (Bandler & Grinder, 1979) that has gained recent interest (Adams & Allan, 2019; Astill Wright et al., 2021a, 2021b; Gray & Bourke, 2015), may work through similar reconsolidation-updating mechanisms as imagery rescripting supposedly does. In line with principles of reconsolidation, this approach starts with a minimal evocation of the index trauma (Lee et al., 2017). Clients are then instructed to imagine their trauma on a movie theater screen from an observer perspective, with the imagined scene starting right before the traumatic event took place (where the client was still safe). At first, clients imagine their trauma memory played forward, then, several times, with increasing speed, fast-backward (up to ≈2-10 s depending on study protocol) to update the chronological sequence of the trauma memory. Although research regarding this innovative ‘rewinding’ approach is still in its infancy (Gray & Liotta, 2012), clinical findings are promising. In line with initial anecdotal findings (Hossack & Bentall, 1996; Muss, 1991 & 2002), several clinical studies report nearly full remission of intrusive PTSD symptoms and high rates of maintenance of treatment success, with researchers emphasizing the application of this imagery approach for patients suffering from high levels of intrusions (Gray & Bourke, 2015; Gray et al., 2019; Tylee et al., 2017). Thus, ‘rewinding’ could be a promising new intervention for treating intrusions; however, no rigorously controlled experimental study has yet investigated the ‘rewinding’ approach, leaving open the possibility that observed clinical gains were due to nonspecific factors like outcome expectancy.

The urgent need for the development of more effective treatment approaches for PTSD has recently been stressed (Olff et al., 2019) and ‘rewinding’ could enhance existing intrusion treatment modules. Compared to currently established intrusion treatment approaches, ‘rewinding’ has several advantages (c.f., Gray et al., 2019). Firstly, in contrast to (prolonged) imaginal exposure treatment, it is a rather brief, cost-effective, and mildly burdensome and distressing intervention, since the core trauma memory needs to be activated for a relatively short time in order to render the method liable (Lewis et al., 2020). Secondly, ‘rewinding’ may bear longer-lasting treatment effects, as reactivation might be long enough for reconsolidation to take place (i.e., making it possible to update fear memories, making them less distressing), but too short for fear reacquisition (i.e., further strengthening the fear memory trace; Alberini, 2005; Merlo et al., 2014; Riccio et al., 2006). Thirdly, in contrast to imaginal exposure and imagery rescripting, clients do not need to narrate the index trauma to the clinician, which provides privacy and data protection and may thus be preferred by some clients; further, this may protect therapists from secondary traumatization. Although advantages prevail, the ‘rewinding’ approach has yet to be established as a standard intrusion intervention module within established cognitive-behavioral therapies for PTSD.

## The Present Study

By using an analogue design, the present study sets out to test the ‘rewinding’ approach in a controlled laboratory setting. It consisted of two experimental sessions: on day 1, participants watched a highly aversive film clip (trauma analogue); on day 2 (24 h later), they either imagined this clip fast-backward (starting with the highly aversive film scene and ending with a scene where the protagonist was still safe), fast-forward (starting with the film scene where the protagonist was still safe and ending with the highly aversive scene), or were assigned to a passive no-intervention control condition. We implemented both a no-intervention passive control condition and a fast-forward control condition to start to dismantle the mechanisms behind 'rewinding' and examine whether its effects rely on replaying the trauma backward, and are not solely based on imaginal exposure, an intervention previously shown to reduce intrusions in analogue and clinical settings (Cusack et al., 2016; Ehlers et al., 2012; Ehlers et al., 2012; Foa et al., 2006). Participants were instructed to report intrusions and associated distress in an event-based manner from day 1–7 in a smartphone e-diary application. We expected imagining analogue trauma fast-backward to result in less intrusion load (number × distress of intrusions) than the passive and fast-forward conditions. Secondarily, we also expected the fast-forward condition to result in less intrusion load than the passive control condition.

## Transparency and Openness

The studies and analyses were not preregistered. The Stan-based package brms (Bürkner, 2017; Carpenter et al., 2017) and R (R Core Team, 2019), both of which were used in analyses (see below), are publicly available, and procedures for their use are well characterized in software documentation. Legally, we are not allowed to share the data of the present study, as no consent of participants for open data was obtained. Research materials and analysis code are available from the corresponding author. This work involved human participants and adhered to the guidelines of the 2013 Declaration of Helsinki. All procedures were approved by the Ethics Commission of Paris Lodron University Salzburg and all participants provided informed consent. We report all data exclusions, all manipulations, and all measures in the study.

## Methods

### Participants

Exclusion criteria were lifetime diagnoses of any mental disorder, serious medical conditions, ongoing psychotherapeutic treatment and psychotropic medication, extensive violent media consumption (> 3 times a week), and low overall vividness of visual imagery as determined by values < 4.5 (“not clear or lively”) on the Vividness of Visual Imagery Questionnaire (VVIQ-RV, Marks, 1995; McKelvie, 1995). We wanted to only include participants with a sufficient ability to visually replay memories in different new ways (i.e., the rewinding and fast-forward interventions).

We aimed to include at least 66 participants. Of the 115 women tested, 6 participants were excluded due to technical problems (N = 5) or dropped out due to high emotional reactivity to the film (N = 1). Of those 6 participants, 3 were excluded before randomization and 3 participants were excluded after randomization as testing had been erroneously continued for them. Further, 23 participants were excluded before randomization as they reported zero intrusions in the 24 h following film-viewing, rendering any intervention inappropriate. Finally, 5 participants were excluded after randomization due to low diary compliance (missing an evening checkup questionnaire > 3 times across the seven assessment days).

The final sample consisted of 81 women (age: *M* = 21.67 years, *SD* = 3.91). Participants were reimbursed with course credit or 50 Euro. All participants provided informed consent; the study was approved by the local ethics committee.

## Materials and Procedure

### Questionnaire Data

Several days before the experimental session, participants filled out the *S*tate-Trait-Anxiety Inventory (STAI, German version: Laux et al., 1981), the Center for Epidemiologic Studies Depression Scale (CESD; Radloff, 1977; German version: Meyer & Hautzinger, 2001), and the Vividness of Visual Imagery Questionnaire (VVIQ-RV, Marks, 1995; McKelvie, 1995).

### Experimental Setup (see Fig. 1)

**Fig. 1:**
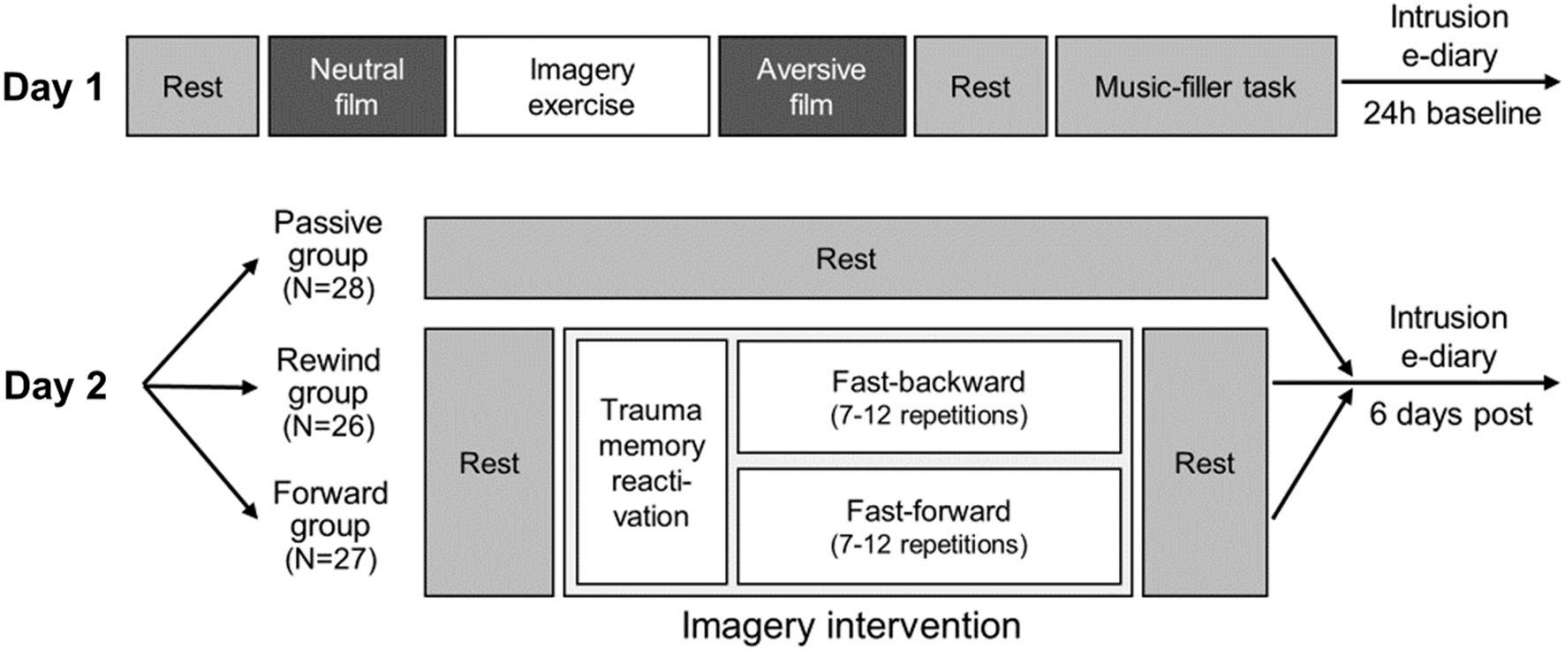
Experimental Procedures (see text for details)

Participants were tested twice, with appointments scheduled 24 h apart in the afternoon/evening (*M* difference time-of-day between appointments≈52 min). Note that the present study focuses on intervention effects; for physiological and eye-tracking data during film-viewing, see Danböck et al. (2021).

**Day 1**. At first, participants underwent a quiet eyes-open rest period (duration: 5–7.5 min^1^). Following, they were informed that they would now practice imagining film scenes. Participants then watched a neutral film clip depicting a police officer coming home after work, following his daily routines (Gröning et al., 2013, duration: 5–7.5 min). They were instructed to follow the film plot as closely as possible, imagining that they would be present in the scene as an observer. Thereafter, participants rated the distress, arousal, and (un)pleasantness associated with the film-viewing.

***Imagery Exercise.*** Subsequently, the experimenter entered the room and sat next to the participant. First, participants were instructed to close their eyes and describe how they felt during film-viewing (reactivation). As soon as reactivation was successful, participants were reoriented to the room by making them aware of their sitting position. Following, participants were instructed to visualize the beginning of the neutral film scene (eyes closed), imagining this scene on an imaginary computer screen. Then, participants were verbally guided through the plot by the experimenter from the beginning to the end (e.g., “*Imagine how the officer walks into the living room and switches on the lights*”). Then, participants practiced imagining this plot in reverse, increasingly fast-paced, with a set minimum of seven and a maximum of 12 attempts. The aim was to reduce the visualization process to three seconds.

***Aversive Film-Viewing.*** After successful training, participants watched an aversive film depicting a rape scene including physical and sexual violence (Rossignon & Noé, 2002; duration 5–7.5 min) which has been proven successful in eliciting intrusions (Arnaudova & Hagenaars, 2017). Note that it was crucial for our later intervention that the film started with a neutral scene (woman at a party) before it turned aversive.

Film-viewing instructions were similar to the neutral film. The film was followed by a 3 min eyes-open resting period and a 10 min music filler task, where participants rated classical music excerpts in order to provide time for the consolidation process to unfold (James et al., 2015). At last, participants rated the distress, arousal, and (un)pleasantness associated with the film.

***Pre-Intervention Baseline Intrusions***. Participants were instructed to report any film-related intrusions in an event-based manner using an e-diary smartphone application until the next experimental session on day 2.

**Day 2.** If participants reported zero pre-intervention intrusion load, they were not assigned to any experimental group and excluded from the experiment. All other participants were randomized to one of three experimental groups, with computerized covariate-adapted assignment considering their number of pre-intervention intrusions, age, and type of compensation (money vs. course credit for psychology students).

If participants were assigned to the ***passive control group***, they completed a 10 min eyes-open resting baseline only.

***Imagery Intervention*.** If participants were assigned to one of the two intervention groups (forward/rewind group), the experimenter sat next to them and instructed them to close their eyes and to shortly describe how they felt during the aversive film-viewing from the day before. If participants did not answer, we asked which scene of the film left the strongest impact on them (reactivation; “*Which scene do you remember most*?”). When the participant`s response indicated a memory reactivation, participants were reoriented to the room by making them aware of their sitting position. Next, they were asked to visualize the neutral beginning of the film (i.e., woman at the party; “*Imagine that you are watching this scene once more on the computer screen. On the screen you can see a still image of the woman standing at the top of the stairs. Can you see the image in your mind`s eye?*”).

If assigned to the*** forward group***, participants should visualize the film fast-forward (similar to watching a DVD fast-forward). This implied that they should start with the neutral scene where the woman was still at the party and end with the scene where the perpetrator brutally hit the woman`s head on the floor. It was emphasized that they should make sure to always end with this scene.

If assigned to the ***rewind group***, participants should visualize the film fast-backward. This implied that they should start with the scene where the perpetrator brutally hit the woman`s head on the floor and end with the neutral scene where the woman was still at the party. It was emphasized that they should make sure to always end with this scene.

For both intervention groups, the aim was to reduce the fast-forward/backward imagery process to three seconds, with a set minimum of seven and a maximum of 12 attempts; participants opened their eyes between each attempt. Both interventions started and ended with a 3 min eyes-open resting baseline.

### Stimulus Presentation

The film was presented on a 23.6″ monitor (positioned ~ 60–65 cm away from participants) using E-Prime 2.0 (Psychology Software Tools, Sharpsburg, PA). Sound was presented via loudspeakers.

### Ratings

Participants verbally rated the distress (ranging from “*not at all*” to “*very much*”, 0–100), arousal (ranging from “*not at all*” to “*very much*”, 0–100), and (un)pleasantness (ranging from “*very pleasant*” to “*very unpleasant*”, 0–100) associated with the films.

### Intrusive Memories

Participants reported film-related intrusions for the rest of day 1 and the six consecutive days in an event-based manner using an e-diary smartphone application (Rattel et al., 2019a, 2019b, 2019c). Intrusions were defined as recurring images or thoughts about the film (Intrusive Memory Questionnaire, IMQ; Zetsche et al., 2009). Only involuntary memories and no deliberate recall should be recorded; intrusions during the night (e.g., dreams, awakenings) were also counted. Each intrusion was rated in terms of its associated distress (0 “*not at all distressing*”—100 “*extremely distressing*”; visual-analogue-scale).^2^ In line with previous publications (e.g., Rattel, Miedl, et al., 2019; Rattel et al., 2019a, 2019b, 2019c), we were particularly interested in intrusion load, i.e., the frequency of intrusions weighted for their distress (frequency × distress), as clinical research showed that intrusions perceived as distressing are those that are primarily linked to PTSD (Steil & Ehlers, 2000). We calculated (1) pre-intervention intrusion load, i.e., the frequency of intrusions in the 24 h pre-intervention (spanning day 1 and the beginning of day 2) multiplied with the accompanying distress and (2) post-intervention intrusion load, i.e., the frequency of intrusions during the remainder of day 2 and the five consecutive days multiplied with the accompanying distress. If no intrusions were reported, intrusion load and frequency were set to zero.

### Statistical Analyses

Using IBM-SPSS-20, one-way ANOVAs were computed to check for pre-intervention group differences in descriptive statistics. Using the Stan-based package brms (Bürkner, 2017; Carpenter et al., 2017) in R 4.0.3 (R Core Team, 2019), we computed two Bayesian regression models to assess group differences in (1) pre- and (2) post-intervention intrusion load. Each time, we entered group as dummy coded variable. Direct comparisons between all groups were calculated and reported. The outcome intrusion load was, similarly to previous work (Franke et al., 2021, 2022a, 2022b), fitted with a hurdle lognormal distribution (Li, Elashoff, Robbins, & Xun, 2011; Tooze, Grunwald, & Jones, 2002) to account for the inflation of zero intrusion load in the data. Hence, the resulting models include two parts where (A) estimates the effect of experimental group on the probability of not experiencing (i.e., zero) vs. experiencing (i.e., non-zero) intrusion load (hurdle part, modelled with a binomial distribution) and (B) estimates the effect of experimental group on the severity of existing intrusion load > 0 (lognormal part, modelled with a lognormal distribution).

We report regression coefficients (bs) and, following the recommendations of Kruschke (2014) and McElreath (2020), 89% credible intervals (CIs), i.e., Bayesian confidence intervals, for all group differences in post-intervention intrusion load. 89% CIs constitute intervals containing 89% of posterior draws. Additionally, we report the posterior probability of each coefficient being in the expected direction, i.e., the percentage of posterior draws being greater (hurdle part; PP_b>0_) or smaller (lognormal part; PP_b<0_) than zero. Effects were considered significantly different from zero if the estimate’s 89%CIs did not include zero.

## Results

### Group Characteristics (see Table 1)

**Table 1:** Group characteristics

	Mean (*SD*)			*p*
	Passive (*N* = 28)	Rewind (*N* = 26)	Forward (*N* = 27)	
Age	21.25 (3.61)	22.62 (4.59)	21.19 (3.46)	0.333
Years of education	14.38 (3.14)	15.15 (3.54)	14.37 (3.18)	0.610
Psychological trait characteristics Trait anxiety (20–80)	40.75 (9.03)	39.31 (9.00)	40.07 (9.40)	0.846
Trait depression (0–60)	13.00 (7.37)	12.65 (6.29)	13.44 (7.11)	0.917
Vividness of visual imagery (1–7)	5.21 (0.81)	5.35 (0.56)	5.48 (0.51)	0.310
Trauma film Distress (0–100)	78.04 (21.96)	81.92 (15.88	73.89 (19.81)	0.329
Arousal (0–100)	74.46 (21.96)	74.62 (23.19)	73.70 (17.35)	0.986
(Un)pleasantness (0–100)	93.21 (7.23)	92.31 (9.72)	87.04 (13.61)	0.070
Number of visualization trials (day 2)		7.42 (1.68)	8.40 (2.12)	0.493

*Note*. Trait anxiety was assessed with the STAI (German version: Laux et al., 1981), trait depression with the CESD (Radloff, 1977; German version: Meyer & Hautzinger, 2001), and vividness of visual imagery with the VVIQ-RV (Marks, 1995; McKelvie, 1995)

The three experimental groups did not differ regarding age, trait questionnaires, and how they experienced the trauma-film. The rewind and forward groups used a similar number of visualization trials during the intervention phase.

### Descriptive Information on Trauma-Film Intrusions

Across groups and on average, participants had a pre-intervention baseline intrusion load of 120.67 (*SD* = 143.89) and a post-intervention intrusion load of 69.92 (*SD* = 207.97). For descriptive purposes, average daily intrusion frequency and distress are provided in Table 2.

**Table 2:** Descriptive information on trauma-film intrusions

Time period	Intrusion frequency *M (SD)*	Intrusion distress *M (SD)*
Day 1 + Day 2 pre-intervention	3.20 (2.53)	36.46 (24.84)
Day 2 post-intervention	0.64 (1.08)	29.34 (26.94)
Day 3	1.00 (1.41)	23.81 (22.77)
Day 4	0.79 (1.24)	28.43 (26.49)
Day 5	0.48 (0.96)	22.22 (23.67)
Day 6	0.32 (0.65)	25.37 (26.19)
Day 7	0.30 (0.80)	20.87 (26.06)

*Note.* Intrusion frequency descriptives were calculated with respect to all participants (N = 81) with intrusion frequency set to zero in case of no intrusion during the respective period. Intrusion distress descriptives were calculated with respect to the participants who had at least one intrusion during the respective period

### Effect of Experimental Group on Pre-Intervention Intrusion Load

As expected, we found no above-threshold group differences in the pre-intervention probability of having zero intrusion load (hurdle part: b_Forward-Passive_ = 0.04, 89% CI [− 1.65, 1.72], PP_b>0_ = 0.52; b_Rewind-Passive_ = −0.75, 89% CI [−2.96, 1.23], PP_b>0_ = 0.29; b_Rewind-Forward_ = −0.79**,** 89% CI [−2.99, 1.16]), PP_b>0_ = 0.27) nor in pre-intervention intrusion load severity (lognormal part: b_Forward-Passive_ = −0.28, 89% CI [−0.84, 0.28], PP_b<0_ = 0.79; b_Rewind-Passive_ = −0.41, 89% CI [−0.98, 0.15], PP_b<0_ = 0.88; b_Rewind-Forward_ = -0.14**,** 89% CI [−0.70, 0.43], PP_b<0_ = 0.65).

### Effect of Experimental Group on Post-Intervention Intrusion Load

Regression coefficients, 89% CIs, and PP of all comparisons are summarized in Table 3.

**Table 3:** Effect of experimental group on post-intervention intrusion load

	Rewind—Passive	Rewind—Forward	Forward—Passive
Hurdle part	b = −0.43 89% CI [−1.41, 0.52] PP_b>0_ = 0.24	b = −0.81 89% CI [−1.77, 0.11] PP_b>0_ = 0.08	b = 0.38 89% CI [−0.50, 1.28] PP_b>0_ = 0.75
Lognormal part	b = −1.15 89% CI [−2.17, −0.10] PP_b<0_ = 0.96	b = −0.45 89% CI [−1.57, 0.66] PP_b<0_ = 0.74	b = −0.69 89% CI [−1.80, 0.41] PP_b<0_ = 0.84

*Note.* Regression coefficients (b), 89% credible intervals (CI) and posterior probabilities (PP) of all contrasts of interest are reported. For the hurdle part of the model, positive b values would denote higher probability of intrusion load absence for the Rewind group (Rewind−Passive, Rewind−Forward) or the Forward group (Forward-Passive). For the lognormal part, negative b values would denote lower intrusion load severity for the Rewind group (Rewind-Passive, Rewind-Forward) or the Forward group (Forward-Passive). For the hurdle part, PP of positive b values (PP_b>0_) are reported. For the lognormal part, PP of negative b values (PP_b<0_) are reported

**Fig. 2:**
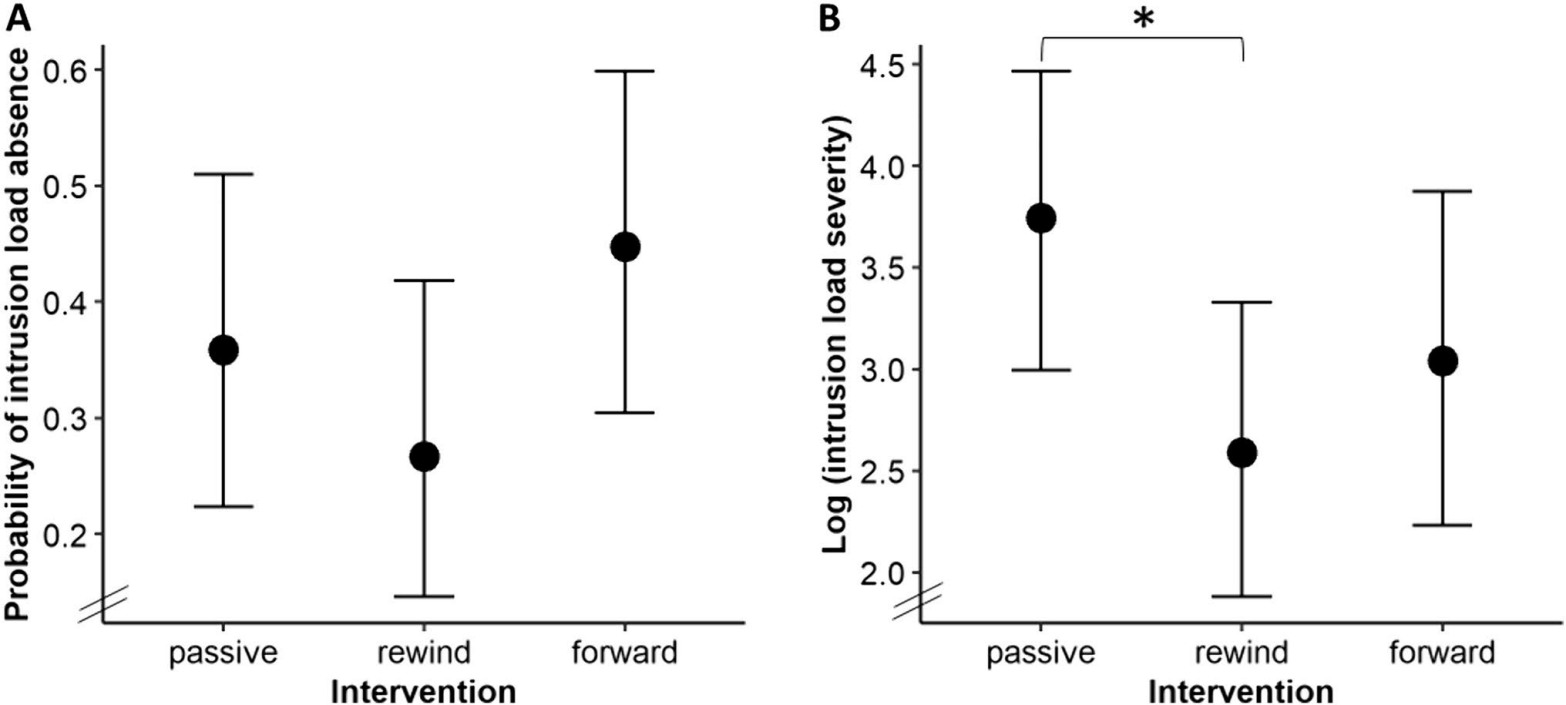
Effects of experimental group on post-intervention intrusion load. Note. Fitted values of the regression model predicting post-intervention intrusion load by experimental group. Vertical lines represent 89% CIs. Panel A depicts effects of experimental group on the post-intervention probability of intrusion load absence (hurdle part). Panel B depicts effects of experimental group on post-intervention intrusion load severity depicted on a base-10 logarithmic scale (lognormal part). *89% CI of difference not including zero.

**Probability of zero intrusion load (hurdle part, see **Fig. 2**A)**. Contrary to our expectations, no above-threshold group differences in the probability of having zero intrusion load post-intervention emerged.

**Intrusion load severity (lognormal part, see **Fig. 2**B).** In line with our expectations, the rewind group reported lower post-intervention intrusion load severity than the passive control group. However, no above-threshold differences were found between the forward and the passive control group and between the rewind and the forward group.

## Discussion

The present study was the first to investigate the proposed ‘rewinding’ treatment approach for intrusive re-experiencing of traumatic events in a controlled analogue setting. We found that mentally replaying analogue trauma fast-backward reduced intrusion load severity during subsequent days compared to a passive control group. Our data did not provide above-threshold evidence for a difference in intrusion load between fast-forward imagination and the passive control group and between replaying the trauma backward vs. forward. Nevertheless, the present findings strengthen the view that, following trauma, ‘rewinding’ could be a promising intervention for reducing intrusive re-experiencing, a core symptom of PTSD (American Psychiatric Association, 2013).

### Underlying Mechanism of the ‘Rewinding’ Approach

Present findings are in line with the idea that intrusions can be reduced by mechanisms of memory updating (Lee et al., 2017; Morina et al., 2017). In line with the idea of reconsolidation-updating (Merlo et al., 2014), the present study used short reactivation of analogue trauma, followed by reorientation to the room, and subsequently imagining the analogue trauma fast-backward, which led to significantly less intrusion load than no intervention. By imagining analogue trauma in reverse, participants mentally replayed trauma from the most aversive film scene (“hotspot”; Ehlers et al., 2002) to a scene where the protagonist was still safe. In doing so, we believe that participants updated their memories in a way that changed the sequence of events (from highly aversive to safe), thereby reducing the affective tone and distressing quality of the memory.

Moreover, as has already been proposed by Bandler (1985), the fast-paced nature of the imagination might have additionally reduced the affective tone of the memory. We propose that fast imagination takes advantage of the narrow window of memory lability and therefore, may not leave sufficient working memory capacity for fear responses to be once again accessed and stored (Miller, 1994). Although our data does not provide above-threshold evidence for an effect of fast-forward imagination vs. passive control, the respective posterior probabilities suggest that fast forward imagination is likely more effective than passive control, providing some support for a general effect of fast imagination. In a similar vein, our data did not provide above-threshold evidence for differences in intrusion load between fast-forward and fast-backward imagination, with also the respective posterior probabilities not providing a clear picture, suggesting a better outcome for fast-forward imagination regarding intrusion load absence but a better outcome for fast-backward imagination regarding intrusion load severity. Anyhow, with the current design we could not disentangle whether the fast pace of the imagination or the direction of the imagination (backward vs. forward) constitutes the effective ingredient. Future studies might include fast-paced and normal-paced backward and forward imagination conditions to examine the specific contributions of imagery direction and speed on intrusions.

Further, in the present analogue study, the fast-forward imagery ended with a very aversive film scene, whereas the fast-rewind imagery ended with a film scene where the protagonist was safe. This alone could have possibly influenced the frequency and distress of subsequent intrusions, as it has been shown that “feeling safe” is an important mechanism when treating PTSD with imagery rescripting (Bosch & Arntz, 2023). Moreover, ending safe could have introduced new, incompatible information to the original fear structure, constituting an important mechanism of exposure therapy (Foa & McLean, 2016). Therefore, future studies should test for differential effects between fast-forward and rewind when both intervention groups end imagery with a safe situation, to further disentangle the mechanisms at play. Finally, one might also examine the necessity of the trauma theme and compare trauma reactivation + fast rewinding of the trauma situation to trauma reactivation + fast rewinding of an unrelated neutral or positive situation.

Present findings match past analogue studies examining imagery rescripting, another intervention technique thought to make use of reconsolidation-updating (Dibbets & Arntz, 2016; Hagenaars & Arntz, 2012). In these studies, imagery rescripting, a method also changing the course of the traumatic event but not necessarily by 'rewinding' it, reduced intrusive re-experiencing compared to several control conditions. One might argue that 'rewinding' could be understood as a specific form of imagery rescripting, where the event is rescripted in reverse order in fast-pace. In contrast to standard imagery rescripting, 'rewinding' would require less input and creative thought from the patient and/or therapist and might thereby be easier to implement during therapy. Altogether, 'rewinding' might constitute a particularly useful form of imagery rescripting especially in patients not willing to disclose much detail about their traumatic event or not being able to come up themselves with ideas how to rescript the event. Future trials might therefore compare 'rewinding' to standard imagery rescripting.

### Methodological Considerations

Using reconsolidation-updating bears several advantages when compared to the traditional extinction mechanism underlying imaginal or in-vivo exposure. Memories updated by means of reconsolidation seem more persistent, as research showed that they primarily reduce the emotional memory component though leaving the declarative memory component intact (Beckers & Kindt, 2017; Kindt et al., 2009); therefore, extinction-based rapid reacquisition, reinstatement, and other return-of-fear responses should be reduced (Gray et al., 2019; Tylee et al., 2017; Vervliet et al., 2013). Moreover, reconsolidation-updating approaches consist of shorter exposures to the trauma stimulus, with one reactivation and few subsequent modification trials being often sufficient to modify trauma memories (Beckers & Kindt, 2017); this could essentially reduce dropout rates (Lewis et al., 2020). In comparison to other analogue studies on imagery rescripting that used same-day interventions (Dibbets & Arntz, 2016; Hagenaars & Arntz, 2012), the present study incorporated a longer, 24 h, trauma-film consolidation window (Acmacher & Rasch, 2017). In terms of experimenter control, it is of great advantage that analogue studies incorporate a consolidation window of equal duration across all participants. However, this rather short window hardly reflects the elapsed time between real-life trauma and clinical interventions; thus, the strength of consolidation differs between analogue und clinical studies, limiting the generalization of present findings, as older memories are more persistent (Alberini, 2011). Though, by successfully applying the ‘rewinding’ approach after a short (24 h) consolidation window, we believe that the present findings suggest that ‘rewinding’ could be a promising, easy-to-implement mental-health intervention soon after trauma, with effective early interventions still lacking (Roberts et al., 2010).

In terms of limitations, generalization of present findings is restricted to (primarily white, European) women; due to already reported sex differences in the frequency and distress of analogue intrusions (Rattel et al., 2019a, b, c), with men reporting lower levels of intrusive symptoms than women (Carmassi et al., 2014), the ‘rewinding’ intervention may work differently for men. In addition, results may not generalize to women from other ethnic identification or cultural backgrounds, and this should be taken into account by future studies. Moreover, the present study did not test for declarative (i.e., explicit) memory recall at the end of the study. We would expect that, as in imagery rescripting (Ganslmeier et al., 2022), explicit (recognition) memory would still be intact, indicating that the trauma-film memory had not been erased but that its negative affective tone and intrusiveness has decreased (Brewin, 2014; James et al., 2015); though, this claim needs further testing.

Moreover, we do not have definite proof that the present findings were in fact driven by reconsolidation, or if ‘rewinding’ merely facilitated the formation of a new memory trace (Brewin et al., 2010); however, we do believe that our reconsolidation-updating protocol reflects previous recommendations (e.g., Schiller & Phelps, 2011). In order to back present findings, a future analogue study may include an experimental condition that mentally replays trauma without previous reactivation. If reconsolidation is the mechanism at play, this experimental group should display more intrusion load than an experimental group undergoing reactivation first. In addition, future studies should test the necessity of a music filler task on day one.

Importantly, it should be noted that rewinding could be a promising intervention module for reducing intrusive memories; however, PTSD symptomatology in most patients is much broader and can be addressed with established cognitive behavioral treatments (e.g., Ehlers et al., 2005; Hoppen et al., 2024). Lastly, we want to note that we used a simplified version of the ‘rewinding’ approach, thereby deviating from current clinical ‘rewinding’ protocols (Wright et al., 2021; Gray et al., 2019). We decided to not incorporate “double visual-kinesthetic dissociation” from the trauma in our study protocol; for this, clients are instructed first to imagine their traumatic experience on a movie theater screen, then floating out of their body to the projector to watch themselves sitting in the theater watching the film from a third-person perspective. Using analogue trauma, we believe that inducing more distance from the film scenes is neither necessary nor possible, as participants have already experienced the depicted trauma from a third-person perspective. Future studies with analogue trauma experienced from a first-person perspective (i.e., Trier Social Stress Test) will have to examine the impact of perspective change during ‘rewinding.’

Unfortunately, when using the trauma-film paradigm, long-lasting treatment effects of the ‘rewinding’ approach can only be assumed, but not tested, as analogue intrusions typically subside within days (Rattel et al., 2019a, b, c). Although analogue studies bear several strengths due to their controlled experimental setting, they do face some limitations. Using the trauma-film paradigm, they lack personal relevance (i.e., participants are watching analogue trauma from a passive, third person perspective) and will never compare with the aversiveness of actual trauma. Yet, initial clinical studies have suggested that 'rewinding' might also be effective for PTSD symptoms after actual trauma (Gray & Bourke, 2015; Gray et al., 2019; Tylee et al., 2017), complementing the findings of our randomized controlled investigation in an analogue setting.

## Review, Outlook, and Conclusion

When considering the present findings, one should bear in mind that the study of reconsolidation updating is still in its infancy (Monfils & Holmes, 2018; Phelps & Hofmann, 2019). Although very promising effects have been published using pharmacological agents in rats (Nader & Hardt, 2009), as well as humans (Schiller et al., 2011), it is yet rather unclear what constitutes an optimal level of fear reactivation, without risking fear reacquisition. Further: how can we test for successful reactivation of trauma memories? How can researchers make sure that memories are in a malleable state, initiating memory updating of the original memory trace compared to forming new competing memory traces (the proposed mechanism at play in prolonged exposure)? How long does it take for a memory to be fully consolidated, so that we can talk about reconsolidation (regarding the present study: was the analogue trauma film memory fully consolidated when reactivating it on day 2? Or did we merely disrupt consolidation? (See Phelps & Hofmann, 2019, for a review on this). To date, there is no satisfying response to most of these questions. As noted in a recent review, it is yet unclear whether reconsolidation is the actual mechanism explaining memory change following novel behavioral treatment approaches (Monfils & Holmes, 2018; see also Astill Wright, Horstmann, et al. (2021) and Walsh et al. (2018) for a systematic review on reconsolidation studies). Therefore, present findings should be interpreted bearing these methodological challenges in mind (Chen et al., 2021; Kanstrup et al., 2021).

Despite the challenges faced when studying reconsolidation, we believe that the present analogue study offers important ideas for future treatment approaches. Present results strengthen the view that distressing intrusions can be successfully reduced by 'rewinding', a short intervention incorporating reactivation of trauma followed by mentally replaying trauma fast-backward. The ‘rewinding’ approach could be a promising intervention for clients suffering from intrusive re-experiencing, either shortly after trauma or in the long-term, and might set the ground for further research in healthy and clinical populations.

## Footnotes

1 Between-participant variation was due to minor experimental setup changes.

2 Note that we also assessed intrusions in a time-based manner each evening. However, we did not further analyze this data and only used it to check for objective diary compliance. See Rattel, Grünberger, et al., (2019a, 2019b, 2019c) for a discussion on different modes of intrusion assessment and objective diary compliance.

## Declarations

### Research Involving Human and Animal Participants

No animal studies were carried out by the authors for this article.
